# Does identity shape leadership and management practice? Experiences of PHC facility managers in Cape Town, South Africa

**DOI:** 10.1093/heapol/czu075

**Published:** 2014-09-11

**Authors:** Judith Daire, Lucy Gilson

**Affiliations:** ^1^School of Public Health and Family Medicine, University of Cape Town, Anzio Road, Observatory 7925, Cape Town, South Africa and ^2^Department of Global Health and Development, London School of Hygiene and Tropical Medicine, 15-17 Tavistock Place, London WC1H 9SH, UK

**Keywords:** PHC facilities, health managers, managerial practice, leadership development

## Abstract

In South Africa, as elsewhere, Primary Health Care (PHC) facilities are managed by professional nurses. Little is known about the dimensions and challenges of their job, or what influences their managerial practice. Drawing on leadership and organizational theory, this study explored what the job of being a PHC manager entails, and what factors influence their managerial practice. We specifically considered whether the appointment of professional nurses as facility managers leads to an identity transition, from nurse to manager. The overall intention was to generate ideas about how to support leadership development among PHC facility managers. Adopting case study methodology, the primary researcher facilitated in-depth discussions (about their personal history and managerial experiences) with eight participating facility managers from one geographical area. Other data were collected through in-depth interviews with key informants, document review and researcher field notes/journaling. Analysis involved data triangulation, respondent and peer review and cross-case analysis. The experiences show that the PHC facility manager’s job is dominated by a range of tasks and procedures focused on clinical service management, but is expected to encompass action to address the population and public health needs of the surrounding community. Managing with and through others, and in a complex system, requiring self-management, are critical aspects of the job. A range of personal, professional and contextual factors influence managerial practice, including professional identity. The current largely facility-focused management practice reflects the strong nursing identity of managers and broader organizational influences. However, three of the eight managers appear to self-identify an emerging leadership identity and demonstrate related managerial practices. Nonetheless, there is currently limited support for an identity transition towards leadership in this context. Better support for leadership development could include talent-spotting and nurturing, induction and peer-mentoring for newly appointed facility managers, ongoing peer-support once in post and continuous reflective practice.

**KEY MESSAGES**

- Managing yourself, as well as other people and relationships, and navigating the wider system are fundamental capacities for managing a Primary Health Care (PHC) facility.
- Nurses who become PHC facility managers and develop a leadership identity appear better positioned to manage their facilities more effectively than those who retain a strong nursing identity.
- A leadership identity is reflected in, for example, managing your own emotions, pro-active engagement with staff and the development of informal relationships that assist in navigating the wider health system.
- The transition from nurse to manager might be supported by actions including: early identification of nurses who aspire to be managers, providing early exposure to managerial demands, induction and peer mentoring on appointment, peer led support after appointment, encouraging continuous reflective practice and being given enhanced decision-making authority.

## Introduction

Better performance to improve access, quality, equity and financial protection is the ambition of most health systems. This desire resonates well with the values at the core of the 1978 Alma-Ata declaration on Primary Health Care (PHC). PHC, therefore, remains a rallying call for health system reform, both internationally (WHO 2008) and in South Africa (NDOH 2013).

Within a PHC-oriented health system, primary care facilities function as the service delivery hub, the critical interface between the population and health services. How the primary care team responds to clients and local population needs is an important marker of whether the health system as a whole has adopted a PHC orientation (WHO 2008). In South Africa, as in many other low- and middle-income countries, PHC facilities are managed by professional nurses (PN)—but surprisingly little is known about the dimensions and challenges of their job, and what influences the way they practise management. There has also been barely any consideration, until recently, about how to support them to work effectively as managers. A recent study is perhaps the first to examine managerial competencies at this level in South Africa (Moyo *et al.* 2013). In this article we present a complementary study, which sought to consider both what the job of being a PHC facility manager (FM) entails as well as what factors shape how these managers do this job. We also sought to explore variation among individuals in their approach to their jobs and the influences over this variation, including professional identity. The ultimate intention was to contribute to wider thinking about how better to support PHC FMs in the study setting, and elsewhere.

The study was conducted as part of the DIALHS (District Innovation, Action and Learning for Health Systems development) project which is being implemented collaboratively with public health managers within one sub-district (Mitchell’s Plain) of Cape Town, South Africa. The wider project aims to strengthen sub-district governance and PHC improvement through an action learning approach. The study reported here was prompted by the sub-district managers’ desire to better support PHC FMs, given their critical role in addressing the multi-faceted demands placed on the Mitchell’s Plain health system.

## The study setting

Mitchell’s Plain is situated to the south east of the city centre. As one of the poorest areas of the city, it is not surprising that the current health status of the population is also poor with problems that reflect South Africa’s quadruple burden of disease (Day *et al.* 2012). In 2011, the total population of the area was over half a million people, twice the 1996 level (http://www.capetown.gov.za/en/stats/Pages/Census2011.aspx, Cape Town Census 2011).

Public health service delivery in Mitchell’s Plain currently falls under the dual authority of the Metro District Health System (MDHS) of the Western Cape provincial Department of Health and local government, the health department of the City of Cape Town (City Health). Reflecting differences inherited from the apartheid era, local government has traditionally focused on providing preventive and promotive services and basic curative services for children through a nurse-led model, as well as environmental health services, and provincial government has focused on adult and curative care, provided through a doctor-led model of care (Barron 2008).

The PHC facilities operating in Mitchells Plain are outlined in Table 1, together with details about their management. All FMs fall under the line management of personal PHC managers who report to either the sub-district manager within City Health or the sub-structure director in MDHS. The larger community health centres (CHCs) offer a wider range of services than clinics, i.e. including emergency unit, curative services for adults (treatment for Tuberculosis (TB), HIV/AIDS and medical and surgical conditions) and women’s health services (basic antenatal care, prevention of mother to child transmission services, obstetric care). One CHC also offers a 24-h service. Clinics largely provide child health preventive services such as immunizations, and women’s health services (e.g. family planning, cervical cancer screening). However, efforts are currently being made to ensure provision of a comprehensive service package in all MDHS and City Health facilities.

**Table 1:** PHC facilities in Mitchells Plain, July 2011

Name	Type	City Health/MDHS	Availability of facility manager
Talfesig	CHC	City Health	Has own manager
Rocklands	Clinic	City Health	Same staff work at the two clinics on alternate days including the manager
Westridge	Clinic	City Health	
Eastridge	Clinic	City Health	Next door to Mitchell’s Plain CHC. Has full time facility manager
Mitchells Plain	CHC and Midwifery and Obstetric Unit	MDHS	Next door to Eastridge clinic. Has own manager. 24-h facility
Lentequer	Clinic	City Health	Has own manager
Phumlani	Clinic	City Health	Has own manager
Mzamonhle	Clinic	City Health	Has own manager
Weltevreden	Clinic	City Health	Has own manager
Crossroads 1	Clinic	City Health	Does not have a manager, manager from Crossroads one visit on alternate days. Has deputy manager on site
Crossroads 2	Clinic	City Health	Has own facility manager
Crossroads	CHC	MDHS	Has own manager
Inzamezabanthu	CHC	MDHS	Has own manager
Mandalay	Satellite	City Health	Has own manager

*Source:* This table was developed using information derived from map of Mitchells’ Plain and key informant interviews.

## Study methodology

### Conceptual starting points

Health management is commonly understood as the behaviours that managers use to improve and sustain organizational performance over time (Vriesendorp *et al.* 2010). These behaviours have two dimensions: ‘managing’ the internal activities of an organization to produce reliable performance and ‘leading’ the staff of the organization and external partners to face challenges and achieve results under complex conditions. Health managers must, in other words, be managers that lead (Vriesendorp *et al.* 2010). Similarly, broader leadership/management theory and organizational research suggests that leadership is the ability to make sound decisions and inspire others to perform well to achieve a common vision (Mintzberg 1975; Zand 1997; Kotter 2001; Hogan and Kaiser 2005), which requires personal (managing self), interpersonal (managing others), informational (communication and analysis) and actional (firefighting and planning) competencies (Mintzberg 2011).

Both sets of literature acknowledge that managerial and leadership practice is influenced by a range of personal, professional, organizational and societal factors (Egger *et al.* 2005; Egger and Ollier 2007; Boyatzis 2008). The identity approach to leadership specifically suggests that a particular influence is an individual’s sense of identity, understood as the collection of personal attributes, values, beliefs, motives and experiences that underpin self-perception (Ashforth 2001) and influence the person’s ways of being, thinking and working (Hill 2003; Lord and Hall 2005; Day and Antonakis 2012). The adoption or development of a leadership identity is, therefore, argued to shape leadership practice (Stets and Burke 2000; Kramer 2003; van Knippenberg and Hogg 2003; van Knippenberg *et al.* 2004; van Knippenberg and van Knippenberg 2005; van Knippenberg 2012)—e.g. by influencing an individual’s ability to motivate subordinates (Lord and Hall 2005) or determining whether an individual adopts self-serving or group serving behaviour (Stets and Burke 2000; van Knippenberg and Hogg 2003), as well as by influencing the personal motivation to learn and develop as a leader (Lord and Hall 2005).

Literature on healthcare professional identity (McConnell 2002) and nursing identity (Gregg and Magilvy 2001), similarly, emphasizes the role of professional identity in shaping practice, and in providing a source of energy to perform work (Okura *et al.* 2013). Nurses’ professional identity clearly, however, focuses on the nursing role, which can be understood as a human and moral practice concerned with providing personalized care to patients (Fagermoen 1997).

A further starting point for this study was, therefore, the idea that nursing professionals appointed as PHC FMs might need to undergo an identity transition—moving away from a nursing identity, focused on clinical practice, to adopt a leadership/managerial identity, focused more on sustaining and improving organizational performance and leading others. This idea was derived partly from the personal experience of some in the study team, and partly from relevant theory. Socialization and organizational researchers have noted, for example, that turning points in life such as moments of role transition force a person to ‘take stock, re-evaluate, revise, re-see and re-judge’ (Ibarra and Snook 2008, p. 5; see also, Ashforth 2001), and may precipitate identity revision as new roles require new skills, behaviours, attitudes and patterns of interactions (Hill 2003). Lord and Hall (2005), moreover, posit a model of leadership development in which becoming a leader is accompanied by identity change, and identity change supports leadership development. They argue that with time and experience, increasingly sophisticated systems guide a manager’s behaviour, knowledge and perceptions, and leadership roles and skills become more central to a person’s sense of self.

### Case study design

Given the exploratory purpose of study, we adopted a qualitative, case study approach focused on the individual PHC FM whose personal practices and experiences are the central concern. Their practices are shaped by multiple layers of context—comprising self (history and experience), relationships (with clients, staff, peers, support staff and managers), specific facilities and communities as well as the organizational setting of the particular sub-district, and of various higher levels of managerial and policy influence. The case study approach was, therefore, also particularly appropriate because it allows a phenomenon to be investigated in its real life context using multiple methods of evidence (Yin 2009).

The eight participating PHC FMs were selected partly due to their willingness to be involved in a study requiring detailed and sensitive interviews. In addition we used pre-set criteria to ensure variation in personal and professional characteristics among the cases selected, allowing exploration of different perspectives or circumstances around the transition from nurse to manager (Flyvberg 2004). These criteria included age, length of work experience as a health practitioner and as an FM, facility type and government authority responsible for the facility. Three of the participating FMs were employed by the MDHS and all MDHS PHC FMs are responsible for the larger facilities (CHCs). City Health employed the remaining five participating FMs. Table 2 summarizes the key characteristics of the selected managers.

**Table 2:** Participating PHC FMs and their characteristics

Age	Time in current position	Years of professional nursing experience	Postgraduate qualifications
35	3 years	5	BSc. Tech. majoring PHC
38	4 years	10	BSc. Tech. majoring PHC MSc., Public Administration
40	3 months	2 year as PN (with 8 years other experience)	BSc. HIV management MA Health management
40	5 years	11	BSc. Tech. majoring PHC
40	2 years	8	BSc. Tech. majoring PHC
40	1 month	7	BSc. Tech. majoring PHC
58	15 years	14	None
62	4 years	20	BSc. Tech. majoring PHC

This table was developed from key informant interviews and initial interviews with facility managers.

All participating FMs were PN by training and had the basic professional training of a Diploma in Nursing and Midwifery. As illustrated in Table 2, the majority (6 out of 8) of facility managers were 35–40 years old and most of them (5 out 8) had been in a PHC FMs position for 2–5 years. Seven of the participants had substantial working experience as PN before they became FMs, whereas one had more varied work experience. All but one also had a Bachelor of Science degree, commonly in PHC, and two had master’s degrees. Two of the participants were functioning as FMs at the time of the study, but were formally employed as operational managers (who spend 50% of their time on clinical duties and 50% on management duties, as delegated by the FM).

### Data collection and analysis

Reflection was the overarching approach of engagement between the researcher and participating FMs. It allowed exploration of individual life stories, daily work experiences, including experience around self-selected management critical incidents, and perceptions of colleagues about the participating FMs’ leadership and management practice. Table 3 provides details of the multiple data collection methods. Individual interviews were recorded and later transcribed. Document review and key informant interviews provided insights into the broader organizational setting and processes in which the managers work. Field notes, a research journal and discussions with colleagues involved in the wider DIALHS project also informed the research process.

**Table 3:** Data collection methods, focus of information collected, and rationale for collection

Data collection methods	Description	Focus of information collected	Reasons for its use
Key informant interviews	Interviews with sub-district level managers	Nature of PHC facility level leadership and management	To inform selection of facility managers to be involved in the study
		Process of initiating engaging with facility managers	
Initial interviews with participating FMs	First interview with participating FMs	Life history—childhood and work experiences	To encourage the facility managers to start talking about themselves/their experiences from childhood
			To start to build trust based relationship
Follow-up interviews	Series of in-depth interviews	Childhood and work experiences, how they became managers, their job and challenges	FMs to reflect on initial information
			Researcher to validate information and get clarification on some events
			Researcher to collect new information
Reflecting on life stories	The researcher synthesized individual stories and presented them back to individual managers	Life history, work experience and how they became managers	To encourage more reflection on personal experiences and to validate information gathered
Reflections on critical incidents	Work-related important events, i.e. crises, achievements, difficulties, challenges or happy moments	Nature of PHC facility level leadership and management	To provide space for facility managers to reflect on critical incidents in their work
		Core leadership and management practices	
Feedback from colleagues about participating FMs leadership and management practice	This was adopted from a 360° appraisal approach. The researcher gathered feedback from facility and sub-district staff and other actors	Leadership and management practice of the participating FMs	To gather perceptions about leadership and management practice of participating FMs
			To encourage participating FMs to reflect on how they are perceived by others
	The researcher synthesized the feedback and reflected on it with individual FMs		
			For FMs to be aware of how their actions and behaviour impact on others
Document review	Job descriptions, key performance areas communication channels and management procedures	Nature of PHC facility level leadership and management	To understand prescribed processes of becoming a manager, required competencies tasks and other expectations
Observations	This involved informal observations in the facility	Interactions between the facility manager and clients or staff	To be familiar with everyday situations in the facility
Validation meetings	Group reflections with all participating managers	Commonalities and differences across participating FMs	To validate experiences of the participating managers. To generate ideas about support for PHC facility managers
Reflections with the wider research team	On a quarterly basis, we had reflections on the wider project including different pieces of research work within it	Methods and process of data collection.	Provided a platform for peer review
		Information being generated	

**Figure 1:**
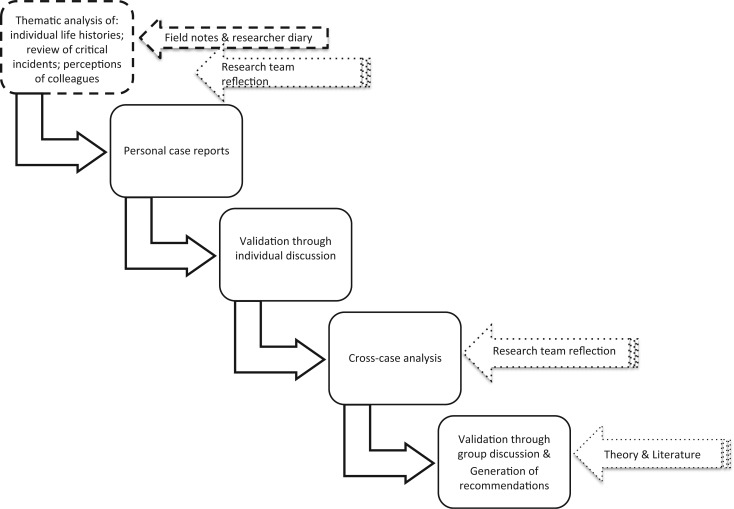
Overall analysis approach.

Figure 1 summarizes the overall analysis approach, which adopted the principles of framework analysis (Green and Thorogood 2004). The preliminary steps of analysis involved the primary researcher reading through all the data sets to get an initial sense of the data and to identify issues to consider further. Data coding used Atlas-ti software, and theme development was partly guided by the conceptual starting points but also allowed for themes to emerge during analysis. Regular interaction with the broader DIALHS research team allowed peer review of the emerging analytic themes. Triangulation across data sets supported the preparation of individual case reports for each participating FM which presented a rich picture of their personal history, managerial experience, managerial practices and working context. Individual discussions with each FM allowed their initial review and validation of these case reports.

The individual reports then, as suggested by Yin (2009), formed the basis of cross-case analysis to identify similarities and differences in experience. This analysis not only considered the conceptual starting points but also allowed for new themes to emerge. The preliminary cross-case synthesis was fed back to allow the group of participating FMs both to interrogate it and to discuss how to offer greater support in future to nurses appointed as PHC FMs in Mitchell’s Plain. Further discussion with the DIALHS research team also informed the analysis. Finally, a study report was prepared and discussed with the sub-district managers.

The study received ethical clearance from the University of Cape Town, Faculty of Health Sciences Human Research Ethics Committee and was undertaken within the overarching DIALHS memorandum of understanding between the two research organizations (Universities of Cape Town and Western Cape), the provincial Department of Health and the health department of the City of Cape Town.

## Findings

### What do PHC FMs commonly do?

Drawing on a combination of job description, interviews and observation, Table 4 outlines the key components of the PHC FM’s job, and identifies the expected knowledge and skills and actual management practice for each.

**Table 4:** Job components, common work processes and routines, and expected knowledge and skills for PHC facility managers

Job components	Expected knowledge and skills	Common work processes and routines	Notes from observations and researcher’s reflections
Managing and monitoring service provision and facility performance	Population health orientation to healthcare service delivery Total population of catchment area and common health problems Number of clients being served by the clinic Calculating monthly, weekly and daily targets for service delivery Drawing up routine monthly statistical reports	Calculating and tracking daily, weekly and monthly targets Submitting routine monthly reports to information officer at sub-district/structure office Carrying out folder audit reviews to assess quality of care Keeping registers for various services Following up clients to return for services Assessing performance of facility staff Attending monthly meetings with managers to discuss progress in meeting service targets and problems affecting service delivery	A central function, and forms a basis of most upward reporting. Each PHC facility is allocated a set of annual prescribed targets,^a^ as performance indicators, which are tracked on a monthly basis
Human resource management	Proactive management of absenteeism Managing interpersonal relationships Role modelling in clinical practice Coaching and supervision of staff members Communication with staff members Directing staff members towards a population orientation to service delivery	Task allocation to staff on daily basis Managing absenteeism and disciplinary procedures Identifying staff development needs Identify need and motivate for additional staff members Dealing with staff complaints about work and personal matters Communicate with staff on one-on-one basis, and through (regular) meetings	The number and categories of staff vary depending on the size and type of the facility, as well as package of PHC services it offers. As CHCs offer a wide range of curative and preventive services, they have a larger size of staff than clinics
Management of medical equipment, drugs and supplies	Knowledge of equipment, drugs and medical supply needs for the clinic that FMs are responsible for Ability to ensure availability of adequate and functioning equipment, adequate drugs and medical supplies at all times Inventory keeping and stock taking	Ordering of drug, medical supplies and equipment Stock taking Develop inventories of equipment Report dysfunctional/missing equipment and supplies to line manager Writing incident reports if medical supplies or equipment missing or stolen Dealing with theft of items by staff, including disciplinary procedures	Function varies between facilities, depending on whether has pharmacist and/or pharmacy assistant or not at all All CHCs for which participating FMs were responsible had a pharmacist. Only one clinic had a pharmacy assistant
Financial management	FMs are expected to know needs and required resources for the clinic Be able to prioritize needs of the clinic and identify resource needs	Attending monthly meeting with line managers to track facility budget (but had little authority over how resources are allocated or used)	Each facility is allocated an annual budget and the facility manager is responsible for controlling the expenditure on some of the budget items, i.e. stationery, staff coffee and tea, staff training
Client and community engagement	Ability to manage conflicts Know importance of community engagement, community structures and role players, other service providers Facilitating community engagements Ensure that there is functioning community health committee	Putting in place a suggestion box for clients Keeping open door for clients Addressing complaints from the community and clients Ensuring functioning of clinic committee and participating in meetings; conducting community profiles	Combines focus on dealing with clients in facility and engaging with community outside facility
Strategic planning	Population health orientation for service delivery and visioning for the future of the clinic Conducting community profiles to identify community needs Prioritizing the community needs and planning for the clinic Linking district and sub-district priorities with that of the facilities	Participate in annual planning processes as invited by sub-district/structure managers Developing key performance areas for self and staff in line with district and provincial priorities and service targets (more formalized in MDHS than in City health)	Planning for the future of the clinic and the health or the population

^a^ Targets are health system performance indicators, which are developed at national and provincial level and passed down to district, sub-district and PHC facility level. Performance of the sub-district then becomes the aggregate of the achieved targets from PHC facilities.

*Source*. Table was developed from consideration of formal job descriptions, interviews with the facility managers themselves, their staff and their managers, and researcher observations within facilities.

Across facilities the main focus of daily practice is on managing and monitoring service provision and facility performance. This entails a range of processes addressing human resource issues, the management of drugs, medical supplies and equipment, control of a few expenditure items and responding to patient complaints. Differences between managers in how these tasks are performed are linked partly to the wider service provision package and larger staff complements of CHCs compared with clinics. In the largest facilities, formal and regular managerial committees bring together those staff nominated as heads of department for different sections of the facility, whereas in smaller facilities all the managerial work is undertaken by the FM and, perhaps, a ‘second-in-charge’ (the operational manager). The larger staff establishment of MDHS facilities also includes a pharmacist who is responsible for daily management of drugs, medical supplies and equipment within the facility, whereas in City Health a pharmacist located at sub-district level supports clinics, with FMs (or clinic-based pharmacy assistants) responsible for ordering drugs and supplies for the facility. In all facilities, a key activity underpinning the other daily management processes is monitoring service performance—involving routine audits of patient records to assess the quality of care being offered, the collection and submission of relevant data to the next managerial layer, and meetings with higher level managers to discuss facility performance in relation to centrally established annual service targets.

Although considerable time is spent addressing patient complaints in all facilities, few FMs currently see broader community engagement or strategic planning, both included in the job description, as key dimensions of their job. Ultimately, therefore, the job of FM as currently practised is primarily oriented towards managing the services within the facility to meet patient needs and reach established service targets. Not surprisingly, therefore, many managers get personally involved in providing services, especially when there are staff shortages.

However, this facility focus runs counter to two key higher-level managerial expectations. First, is the expectation that FMs will adopt an outward focus, moving beyond the facility doors to consider how to improve the health of the community. The central task of facility management is

… *about how to get staff at facility level understand that the health service delivery concept is about community health … So that’s what I am expecting from facility managers that the leadership and management that happens at facility level is for population’s health, not just for the patients who come to the facility.* (KI01)

Second, the expectation that FMs will pro-actively manage their staff and others to ensure that services are provided, rather than stepping in to offer services themselves: ‘one as a manager has got a team, yours is delegation with responsibility and accountability … instead of doing things themselves they have to role model to the team and let the team do the service provision …’ (KI03).

Managing people and relationships, as part of all activities not only formal human resource management processes, is, indeed, the primary demand of daily managerial practice—as illuminated by the set of 32 critical incidents the participating FMs themselves identified over a 4-month period, varying from one to eight per manager. The vast majority of these incidents were linked to managing other people, be it staff or clients. Seventeen incidents related to inter-personal conflicts among staff, coping with absenteeism and dealing with issues of staff personal problems, staff misconduct, including theft of supplies, and five incidents focused on dealing with client complaints and managing clinic committees. A further six incidents focused on managing the overall shortage of staff in the clinics, mostly leading managers themselves to get involved in providing services, two were about dealing with the shortage of drugs or other supplies, and two more were about self-management (e.g. trying to control one’s own anger).

Two extracts from notes taken during discussions with the participating FMs about these incidents illuminate how relationship issues affect daily work:

*In one facility, two nurses were absent, one was on sick leave and the other went for training. The manager called the remaining three nurses and explained the situation for them to share the day’s tasks accordingly. One professional nurse who was allocated for maternal and child health services did not like how the tasks were divided amongst themselves. She just took her handbag, started shouting and she left the clinic. She did not report on duty for three days and was not picking up her phone. She underwent a disciplinary hearing when she reported back on duty.* (FM03)

*In another facility, a client complained that she was not treated well by a doctor, the doctor refused the allegation and it was difficult for the manager to address the issue given the need to maintain a working relationship with the doctor*. (FM06)

Discussion of these experiences with the managers showed that they felt that the everyday management of relationships, and themselves in relation to others, was their most difficult challenge—and one necessary for each formal job component. Similarly, staff within the facilities see giving direction to staff, coaching and communication as important components of the FM role:

*When it comes to helping out when there is staff shortage, the manager is good at that but maybe she also needs to be coaching us and training us so that we can improve our clinical skills … .* (a staff member at FM06’s clinic)

*I liked the meeting today; it is the first staff meeting with our manager since the manager started working at this clinic. It’s a good start but she needs to continue, we need more of these meetings, so that we can discuss problems in our clinic. The manager needs to lead the meetings and give us direction on how we should be working in this clinic … .* (a staff member at FM03’s clinic)

### What common factors drive the ‘facility focus’ in the practice of FMs?

A combination of two key forces seems to drive the dominant facility focus of the FMs participating in this study: professional training and wider organizational and systemic factors.

First, all the managers trained as nurses and value their clinical expertise: ‘… even if you are a manager you are first and foremost a nurse …’. (FM09). They are driven by their commitment to caring for people or saving life and, for many, clinical work offers greater job satisfaction than management:

… *you see a patient who is not able to walk, to talk, to eat or is weak and frail and then you do all you assessments and treat that patient, next time that patient comes back to you walking and talking, it makes you feel good and that’s what makes me happy as a nurse. But as a manager you don’t see the results immediately*. (FM02)

*I always put myself in the shoes of the client. I don’t think making managerial responsibilities a priority over clinical duties is right, when I know that I am able to meet client’s needs, whether that means that I have to work as a clinical person*. (FM06)

As a result, it can be an easy decision simply to step into the service provision role when the need arises, particularly in smaller facilities. Facility staff only add to the pressure to do clinical work, criticizing managers when they do not engage in service provision; and managers themselves see role modelling clinical care for their staff as an important part of their job: ‘As a nurse manager, one is a role model to colleagues and is therefore expected to demonstrate that by actions/doing’ (FM08). Having clinical expertise simply makes it easier for managers to understand protocols and procedures and interpret and communicate them to their staff, as well as to supervise staff in relation to their clinical work.

The second drive towards a facility focus is the broader organizational and system environment. The managers experience service delivery targets, for example, as driving them to focus on service delivery inside the facility:

*… and at the end of the day, targets seem to be the uppermost thing on top of everything*. (FM03)

… *mostly it is the statistics that matter … and the thing is I have to reach the targets and here are the people, because if I don’t do it they will go to the nearest clinic, so let me do them to reach the stats … *. (FM06)

*the challenge is that you need to meet the targets and if you don’t assist clinically you are not going to meet the targets*. (FM05)

The increased workloads experienced over recent years also focus managers on the facility rather than on the community at large:

*The sheer burden of people coming into the facility has increased hugely over the years, which means that a lot of the things that nurses used to do like home visits one can’t do any more. So it has created a narrower scope in a way and also that today people are sicker than 20 years ago. The burden of disease is so overwhelming … *. (FM07)

Other dimensions of workplace experience are also experienced as disempowering. As nurses the managers sometimes feel subject to professional power imbalances that favour doctors, e.g.:

*the most difficult of all people to manage are doctors and pharmacists; they think they are on top of everything because of ranks. They do what they please and they don’t want to follow protocols … every time I have to check if she is on duty or not … she never fills in leave forms.* (FM08)

Managers also feel disempowered by the limits on their authority (e.g. in budget control, Table 4) and by bureaucratic procedures: ‘if you want to discipline somebody, it gets to a point where you have to wait for somebody from the [higher management level] who doesn’t know what’s happening at the facility and the process takes so long’ (FM09). Although City Health managers were delegated authority for handling disciplinary processes during the study period, they felt that they were given too little support to understand the new task and were initially very uncomfortable with the new responsibility. Overall, there is a common feeling that there is:

*… no space to be creative at all. If you want to be creative then you have to use some resources, you know like if you want to have a teambuilding for your staff members, you have to encourage the staff members to fundraise. You want to give your staff members certificates, if you want a printer that is coloured, I am telling you, you will need to write an essay to say why you need a printer that is coloured … *. (FM07)

Finally, managers bear the brunt of a combination of too little policy guidance on some issues and too many policy instructions in other areas. Confusion about the policy guidance on facility committees, for example, underpins the limited emphasis given to community engagement in their daily practice (Table 4). On the other hand, the many policy initiatives linked to expanding the PHC package offered in facilities, each introduced without additional resources, only adds to workloads and job demands.

### Are there variations between managers in how they practice their job?

Despite the common ‘facility focus’ of managerial practice, closer examination of experience across the participating FMs reveals important differences between individual in key dimensions of their leadership: in how they managed themselves and their relationships with others, how they responded to events and people in the facility, and how they navigated the system in which they worked (Mintzberg 2011).

Three FMs (FMs 03/05/07) present a stronger sense that clinical roles and expertise are only one element of their job compared with the other five participating FMs, who more strongly focus on patients and clinical work. FM03, FM05 and FM07 all feel that nurses have a range of roles, such as service provider, teacher, researcher (FM03), and see themselves as adopting the role of manager (FM03) or teacher, supervisor and mentor (FM05). In addition, they see clinical expertise as useful in their managerial roles because it assists in supervising and training staff (FM03 and FM05), assessing clinic performance (FM03) or being able to advocate for patients within the facility (FM07). However, all three commented that they only rarely get involved in service provision:

*Since I started working … as a facility manager, I have never worked as a nurse (service provider), you know when you are being trained as nurses, we are told that we have different roles, some are managers, others are researcher, teachers, advocators, service providers, and one chooses a dominant role to play. The basic nursing training is very important because it helps you to do whatever role you choose to play … *. (FM03)

*… first and foremost I am a nurse, that’s why I am able to supervise my staff, I also organize internal training like if I see that professional nurses are not following procedures or they are not able to do certain things, I teach them how to do clinical work. First, I demonstrate how to do it and then I ask the nurse to do the same thing whilst I am observing and then I give feedback … *. (FM05)

*… my role is to delegate and follow up with operational managers, not to provide services, maybe only when there is a problem. For example if a nurse did not provide a patient with some diagnostics test results or did not speak nicely to a client then I intervene … .* (FM07)

Differences in their approaches to management were also reflected in how managers were experienced by colleagues and in their interactions with other people (Table 5). In contrast to the other FMs, FMs 03/05/07 were all experienced as being better able to contain or manage themselves, not letting their emotions affect their work, and as managing with and through people. Nonetheless there were differences among them: FM03 knew every staff member and supported them emotionally; FM05 was more instructional in approach, expecting staff to comply with set standards of performance; FM07 worked as one removed from the facility staff, working through other managers and managerial processes.

**Table 5:** Feedback from colleagues about the participating FMs’ leadership and management practices

Participating FM	General description (views from self and colleagues)	Interactions with people (views from participating managers, staff and supervisors)
FM03	An emotionally stable person, had past experience in managing projects and clients.	Gives clinic staff an opportunity to contribute to problem solving and decision making. Knows each of the staff members as individuals including their personal problems. Praises those that perform well and encourages those that seem to be struggling
	Values people’s contribution and team work	
FM05	Strong minded person who has a ‘can do’ attitude and is capable of dealing with challenges in the clinic	Staff members have mixed views.
	Is persistent and has a handle on everything going on in the clinic to ensure that nothing goes wrong	Some feel she brings change and improves performance of the facility, brings staff together through social activities eg. celebration of birthdays, condolences for those that lose their loved ones.
	Wants clinic staff to comply with rules and regulations	Some feel she does not listen to others, wants things to be done in her way and wants others to follow instructions
	Sets a very high standard of performance	
FM07	Always in the office and staff come to the office when they need anything.	Interacts more with operational managers and less with clinic staff members. Does most of the communication through operational managers or group meetings
	Calls staff to office if there is need to communicate anything to them or calls a meeting with individuals or groups of staff	
		Staff members feel sees self as the one in-charge at the facility and doesn t want to take other people's ideas.
	Delegates most of the responsibilities to operational managers and focuses more on administrative role, i.e. approving holidays and training, attending meetings and addressing staff and clients’ complaints	
FM02	Described as someone who talks with people through meetings mostly.	Staff members feel deals with staff challenges inappropriately.
	Does what is expected of her, and expects the same from others. Does not understand why people need to be pushed to meet expectations.	Has difficulties in managing frustration when staff are not meeting expectations
FM04	Colleagues think feels isolated from fellow FMs. Feels staff members don't listen to her and feels she can't cope with rapid changes in the health system	Is not in contact with fellow FMs and relies on people to tell her what’s being communicated through emails
		Staff members feel does not communicate to them and distances self from them
	Does not strive to bring change but works to maintain status quo	
		Line managers feel is finding job difficult
FM06	Is aware that people say does not interact with others in the way that they expect	Staff feel gets pressure from supervisor and works hard to meet the demands.
		Staff do not find it easy to interact, and feel distances self from them and can be irritated by them
	Feels people don't appreciate what does and as a manager doesn't need to be friendly	
FM08	Is seen as a quiet person, who focuses on her work rather than interacting with people	Is a quiet person and focuses on work
		Even when a staff member is misbehaving, does not address staff issues directly, instead reports to supervisor
	Does not speak a lot about self	
		Is very good at patient care and organising the facility but doesn't like confrontation
FM09	Seen as a good manager because of participation in clinical work	New in the facility, so clinic staff little experience

Closer examination of managerial responses to self-identified critical incidents also allowed further insight into the differences in practice between these two groups of managers. As summarized in Table 6 we grouped these incidents into the categories of managing self, managing relationships, managing the system (and specifically, human resource and drug supply issues), and then compared the responses between the two groups of managers.

**Table 6:** Variation in responses to critical incidents

Category of leadership and management function	Situations/critical incidents observed	Responses of FM03, FM05, FM07	Responses of FM02, FM04, FM06, FM08, FM09
Managing self	Bringing or presenting self to others	Confident in their role as managers	Low self-esteem in dealing with other professionals, i.e. doctors and pharmacists
	Anger and frustrations	Acknowledge the anger and frustrations, work as normal and aim to address the cause if possible	Disengage and withdraw from everybody and sometimes from work
Managing relationships	Interpersonal staff conflicts	Address interpersonal conflicts actively, i.e. confronting difficult staff	Avoid dealing with interpersonal conflicts amongst staff and when relationships are severely impacting on service delivery engage line manager to settle staff conflicts or approach difficult staff
	Conflicts between staff and clients	Bring both parties together and mediate discussions to address concerns and conflicts	Avoid addressing conflicts between staff and clients, afraid of offending the staff if on the wrong side
	Complaints from the community and clients	Actively address individual client complaints that get reported	Address complaints from individual clients
			Complain about the difficulty of dealing with clients’ complaints
		Participate in community health committees to explain challenges that facilities face	Rarely participate in health committee meetings
		Make effort to have a functioning community health committee	Does not make effort to strengthen a dysfunctional committee
Managing human resources	Human resource gap due to absenteeism and leave time	Share the HR gap with those on duty to share tasks amongst themselves	Ask for extra staff from other clinics
			Do the work of a health provider to fill in HR gap
		Ask for extra staff from other facilities	
		Alerts line manager of predictable HR shortage and ask for replacement	
	Difficult staff and difficult conversations	Handle difficult staff and conversation well and report to line manager for record keeping	Avoid difficult conversations and request supervisor to manage
			Publicly criticise staff members when doing something inappropriate
		Set routines and reinforce standard practices	
		Immediate appraisals and being visible most of the time	Some try to initiate dialogue but find it difficult to manage without supervisor
	Communication with staff	Conduct regular meetings with staff to talk about concerns and how to address them	Conduct ad hoc meetings in response to crisis
Managing drugs, medical supplies and equipment	Shortage of drugs and medical supplies	Delegate to others to other staff members to borrow from other facilities	Phone around and go to other facilities to borrow drugs and medical supplies
Working around established work processes and systems	Delays in procurement of drugs, medical supplies and equipment	Insist and get what they want	Complain about procurement and staff recruitment systems
		Establish relationships with procurement personnel and follows up with them directly	Follow established lines of authority and communication
	Delays in staff recruitment		

#### Managing self

From these experiences, it seemed that FMs 03/05/07 were more confident in their role as managers than their colleagues, and this influenced their approach to their job. For example, FM05 demonstrated firm leadership in the face of difficult staff situations. In one incident staff organized a meeting to discuss challenges in the facility, but FM05 stopped the meeting because she had not been made aware of it and established the principle that such meetings should be organized through her and with consultation on the agenda. In another incident, when the pharmacy assistant ordered drugs, medical supplies and equipment without consulting her, FM05 instructed that as the accounting officer for the facility, she should approve all pharmaceutical orders, despite the pharmacist being labelled as the most difficult staff member to work with. In contrast, FM06 often stepped into the service provision role, reporting that when there are staff shortages it is difficult for her as a nurse, ‘someone with capability to help the sick to sit in the office while patients are staying long on the queue’.

The importance of being confident in your role as a manager was made clear during discussions with facility staff as well as reflections with the managers, and also reflects individuals’ self-perception. The managers repeatedly noted that seeing yourself as a nurse makes the work of being a manager difficult, as you are less able to lead other health workers. As FM07 noted, to be a manager you need ‘to see yourself as a manager, walk and talk like a manager’.

These three managers also appeared more able to contain how their emotional state affected their job. Whilst acknowledging frustrations and personal problems, they tried to work normally or, where possible, took action to address what was bothering them. In contrast, the other five FMs were more likely to disengage from colleagues and sometimes from their work when they were frustrated, or got angry with their colleagues (see Table 5).

#### Managing relationships

Self-management only forms part of the ‘dynamics of management’, at the heart of which is working with, and through, and for people. Managing relationships was seen to underpin all other managerial tasks and functions, such as monitoring service provision and facility performance, human resource management and management of medical supplies, equipment and drugs. FMs manage conflicts between health providers and clients as well as amongst health providers. They must also have difficult conversations with staff about many things, such as poor service delivery, stealing facility property, absenteeism or inappropriate behaviour towards clients and fellow staff members. FMs have to resolve relationship issues at facility level many times before they become disciplinary issues subject to formal disciplinary processes.

And again here, differences in management practices were noted between the two groups of managers. FMs 03/05/07 more actively addressed relationship issues amongst the staff or between staff and clients and only reported to their line manager or when there was need for a formal process, such as a disciplinary process. For example, FM05 told staff members that, ‘I am the ceiling (the end point of everything) and everything ends with me except where there is need for further action at the [next] level’. Her staff members acknowledged that, ‘the manager knows everything that is happening here and whenever there is a problem of any kind, if it gets reported to the manager, the manager finds ways of solving those problems’.

In contrast, the other participating FMs were seen avoiding, or were reported to avoid, addressing relationship problems between staff and clients or amongst staff members, sometimes because they wanted to maintain relationships with colleagues. For example, FM06 was faced with a situation where a client complained about a doctor and the doctor said that the clients’ claims were not true. She felt ‘that was a difficult situation where I did not know what to do, whether to listen to a client or a fellow health professional. In the end, I just listened to the doctor because he is a colleague and we still need him to provide services to other clients’. Faced with a conflict between a doctor and a nurse in her facility, FM04 reported the matter to her line manager for action, noting that ‘at least when the line manager is here people take the discussions seriously and they listen’. In general, FM04 did not like confrontations with people and preferred to draw her line manager into resolving conflicts.

#### Managing in the facility

Two key areas of focus of daily management in every facility are human resource procedures and managing drugs, medical supplies and equipment. Several clear differences were noted between the two groups of managers in how they handled these issues.

In tackling staff shortages in their facility, for example, FMs 03/05/07 were more likely to discuss the situation with their staff and share out the workload, rather than themselves provide services. Different approaches were also noted in the managers’ approach to handling difficult conversations—about reporting late on duty, absenteeism, not following standard operating procedures, refusing task allocations at work, using work hours for personal activities or conflicts with clients. FMs 03/05/07 tended to deal with misconduct immediately, setting routines and reinforcing standard practices—and just being visible all the time. Other managers were much less likely to confront these problems head on and more likely to get angry with their staff. For example, FM05 established a routine that staff members should inform the manager directly when absent due to illness rather than informally sending messages through colleagues. FMs 03/05/07 also regularly conducted staff meetings, seeing them as an opportunity to identify and address problems at work together, and share communication from higher managerial levels. The other managers often felt that they did not have time for these meetings, and so conducted them less frequently. FM08 noted ‘It is not easy to schedule staff meetings, simply because of time’ and FM02 and FM06 noted that they talked to individuals when necessary rather than having staff meetings.

Finally, FMs 03/05/07 were more likely to assign another staff member to manage their facility’s drug supplies and supporting these staff to deal with shortages (providing a car and a driver to collect extra stock, for example). In contrast, other managers chose to deal with problems themselves (FM04 and FM02) or called in their line manager to solve problems (FM07 and FM08).

#### Navigating the system

In any context, managers also have to navigate and learn to work around the established procedures and management systems (Mintzberg 2011). Of particular relevance here are, again, the systems of managing human resources and drugs, medical supplies and equipment.

FMs 03/05/07 pursued what they needed by establishing direct informal relationships with procurement and supply chain staff as well as human resource department personnel. For example, FM07 wanted to establish a patient’s shelter but initial requests were met with negative responses. The manager then bypassed the line manager and negotiated with the director of procurement, collected some cost quotations and proved to the procurement and supply chain personnel that the facility’s budget would accommodate the expense and finally the shelter was constructed. Although HR recruitment processes take a long time, FM03 negotiated for an urgent recruitment of two extra PN, noting that as a manager you need to have informal networks to get things not only done, but done faster: ‘for some things, you just need to know who to talk to’.

In contrast, the other participating FMs were more likely to complain about procedural delays and were commonly observed as relying more on the formal, and sometimes ineffective, communication channels. FM08, for example, submitted an order for lamps for delivery rooms to the procurement office. Three months later she reported to her line manager that she still had not received the order. Due to other commitments, the line manager took about 5 weeks before she followed up with the procurement office. Then, it took another 2 months before the delivery lamps were supplied: ‘It takes way too long for things that you order to be processed; one has to follow up so many times with the procurement office and our manager at the sub-district office’ (FM08).

### What explains the variations in managerial practice among participating FMs?

#### Identity transition

Examination of the differences in how the two groups of managers see themselves and approach their jobs appears to suggest that FMs 03/05/07 have established a stronger leadership identity than their colleagues, even whilst inevitably retaining some sense of nursing identity. Indeed, they self-identify their roles as being about more than clinical practice, demonstrating greater confidence in the managerial role than their colleagues. In addition, the characteristics of their managerial practice are those that reflect what is expected of health managers that lead (Vriesendorp *et al.* 2010) and of leadership, more broadly (e.g. Kotter 2001). They are, for example, pro-active in addressing facility problems and strengthening facility services, and take steps to manage staff and relationships. In contrast, their colleagues’ primary attention remains more firmly, if not entirely, focused on nursing care and they appear less able, as is needed in leadership roles, to contain their emotions, manage relationships and address non-clinical challenges in their facility. Perhaps, therefore, FMs 03/05/07 can be said to have begun an identity transition from nurse to manager/leader.

But what shapes whether or not this ‘identity transition’ is initiated when nurses become FMs? The experience of the participating managers suggests three influences.

#### Childhood experiences

Conger and Ready (2004) argues that the foundations of leadership and management qualities, such as self-confidence, achievement, drive, communication skills and interpersonal competence, may be formed principally in a family environment. Experiences in school and college shape one’s career interest which becomes an arena in which an individual exercises management and leadership roles.

From the life histories of participating FMs, (see Table 7) childhood experiences seemed to influence the way those with an emerging leadership identity approached their job, as well as their aspirations and achievements. For example, the way in which FM05 was treated both at home and in school led FM05 to adopt a ‘can do’ sort of approach to management, being persistent, dealing with unco-operative staff members and community members. She noted herself:

*Even though my childhood, growing up in the village and school experiences were not good, I think those hard experiences made me a strong person today because instead of grumbling about the problems in this facility I am always on my toes to deal with any situation, whether it’s the staff or patients*. (FM05)

FM03, meanwhile, had parents who encouraged her to work hard in school, and to aspire to achieve in life, ‘I would say that it is mainly because of my father for me to be where I am today, he encourage us the children to aspire more and work hard in school to be somebody in the community’.

**Table 7:** Variation in factors influencing the transition from nurse to manager, by participating FM

Influencing factors	FM03	FM05	FM07	FM02	FM04	FM06	FM08	FM09
Childhood experiences (family, and school environment	A big family	Difficult school experiences	Grew up in difficult community	Grew up in protective homes	Lost mother at young age and grew up with auntie who was a nurse and supported her to be a nurse	Grew up in protective family environment	Only child in the family and used to be in church youth club helping elderly and sick people	Grew up with grandmother because parents were working far from home
	Financial constraints on education	Challenging home environment	Family had financial challenges	Mother was a house wife		Raised by a single parent who worked hard to provide for her children and encouraged them to work hard		Home was close to a big hospital and admired nurses in their uniforms
	Influence of parents to achieve and aspire more in life	As a child used to receive gifts from nurses	Unsupportive school environment	Wanted to be educated and be able to earn a living				
Aspirations	Wanted to be a doctor	Wanted to be a lawyer.	Wanted better living standards	Wanted to be a nurse	Wanted to be a nurse	Wanted to be a nurse	Wanted to be a social worker	Wanted to be a nurse
	Wants a high-level management job	Wanted to be PHC facility manager	Wanted to be a lawyer	Became manager because wanted a fixed term job and as an alternative job to service provision	Accepted a management job because it was a promotion	Did not want to be a manager, was encouraged by line manager to apply for the post	Does not want to be a manager	
	Wants to pursue a Doctor of Philosophy	Wants a high-level management position. Wants to do Master of Public Health	Wants a high level management job				Is a manager because was promoted	Does not want to be a manager but sees management as a career development path
			Wants to pursue PhD in public administration					
Previous experience when taking up managerial responsibilities	Worked as second in-charge at hospital level and as research co-ordinator	Worked as operational manager, senior nursing officer and acting FM	Worked as operational manager and second in-charge	Worked as PHC service provider	Worked as a second in-charge	Worked as PHC provider only	Worked as PHC provider only	Worked as operational manager
Support for leadership and management development	Learned through experience. Did a management course	Learned through experience and received orientation from human resource department after had already taken up a managerial position	Learned through experience and did not receive an induction when took up a managerial position	Learnt through experience and learnt a lot by asking previous FM. Received induction after had already taken up a managerial position	Learnt through experience and did not receive an induction	Learnt through experience and did receive orientation by HR department long after had taken up a managerial position	Learnt through experience and did not receive an orientation	Learnt through experience and did not receive an orientation
	Received orientation from sub-district managers							

Family environments (culture, norms, beliefs, practices, values and ways of interacting) also influence the way FMs interact or relate to colleagues, respond to crisis, personal attitudes, and how to manage their own emotions. For example, FM02 and FM06 grew up in a protective family environment, and still find it difficult to interact with staff:

… *my parents were very strict and I grew up in an enclosed community, we were not allowed to go to other communities, the only homes we could go to were our relatives homes that’s why I don’t like people fighting, violence … .* (FM02)

*growing up as a child, I was not allowed to go to other communities, after school I was playing with my sisters. My schedule was also busy because after school, I was taking swimming classes, music classes and playing hockey.* (FM06)

#### Personal aspirations

Defining aspirations as imaginations of possible selves, Markus and Nurius (1986) argued that aspirations play a key role in identity change because they serve as motivational devices shaping personal responses to evolving opportunities. Aspirations clearly shaped the emerging leaders. FM05 resigned from one government authority to move to another to improve her chances of being promoted to a managerial position. Although discouraged by teachers from being a lawyer, FM07 was now pursuing managerial aspirations in nursing. All three of these managers had also not seen themselves as nurses when they were children, but rather as lawyers/doctor, and all noted they only became nurses because it was financially affordable and they saw it as a stepping stone to other career paths.

In contrast, four of the other five managers had aspired to be nurses from a young age. They had not, moreover, particularly sought out managerial positions. Instead they became managers as it was the only promotion opportunity available to them or a career development step (FM02, FM04, FM08 and FM09), or because they were advised to do so by their line manager (FM06). None expressed particular interest in advancing further up the managerial ladder.

#### Prior managerial experience and support for leadership and management development

Following usual MDHS/City Health practice, all participating FMs became managers through a formal selection process. Appointment is open to both internal and external candidates, but most appointments are internal. The participating FMs described the process of becoming a manager as similar to becoming a parent—in that no one tells you how to be a manager and instead you learn to be one by doing the job.

Although most managers had pursued postgraduate training in PHC, they felt that it offered little support for managerial roles—it did not address how to manage everyday crises in the facilities or how to apply the knowledge learnt to real life situations. Two of the three FMs with a stronger leadership identity had completed Master’s qualifications, but this may reflect their aspirations more than signalling the degree’s usefulness for management.

Little other formal support had been provided to these managers before or after their appointment. However, although not a requirement for appointment, the three FMs with an apparently stronger leadership identity had some prior managerial experience which they had found helpful, compared with only two of the remaining five managers. As FM05 noted, ‘I think being a second in-charge or acting facility manager helps in that one gets to be exposed to some of the management responsibilities and challenges’. Recently there have been some efforts to improve support on first appointment with line managers offering briefings, and City Health, for example, supporting a group mentorship process for all managers.

## Conclusion

We set out to explore what management of PHC facilities entails and what factors influence it, drawing on case studies of managers based in Cape Town, South Africa. The findings and analyses were generated through careful and deliberate approaches, with built-in steps of reflection and validation with respondents, peers and theory. Nonetheless, our study is exploratory in intent, generating ideas for further consideration.

The experiences presented show, first, that the job of being a PHC FM in this context is currently dominated by the formal tasks and procedures focused on clinical service management within the facility. However, these managers are also expected to implement a set of more strategic tasks linked to understanding and addressing the population and public health needs of their surrounding community. All job dimensions are, moreover, underpinned by the critical, but less visible, tasks of managing with and through others, and in a complex system, which themselves require management of self. These experiences demonstrate that PHC facility management is not primarily a mechanistic or administrative function, entailing efficient implementation of predesigned roles, tasks and instructions, but is instead a dynamic and strategic process occurring in conditions of uncertainty (Kotter 1990; Uhl-Bien *et al.* 2007; Mintzberg 2011; Day and Antonakis 2012). The necessary management skills extend, therefore, beyond clinical skills or the operational skills of budgeting and planning, for example, to include the so-called ‘soft skills’ of leading organizational change, communication and motivating others (Brinkerhoff and Klauss 1985; Moore 1995; Mintzberg 2011).

**Figure 2:**
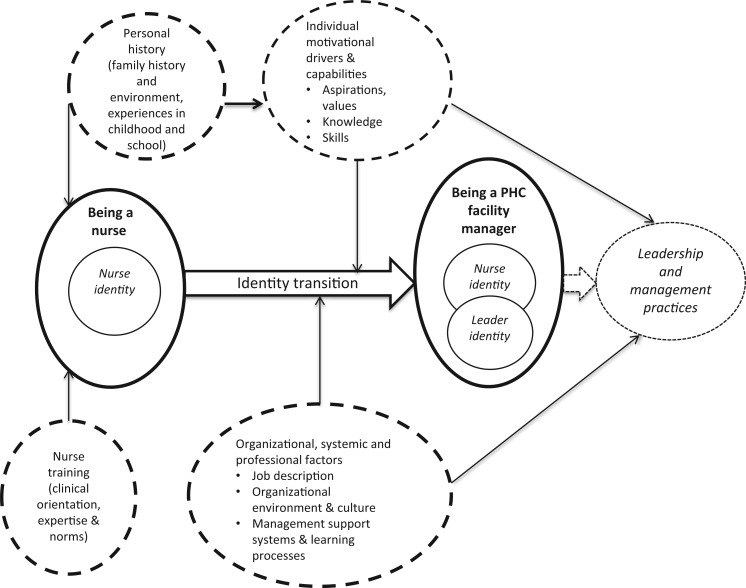
Conceptualizing the factors influencing PHC facility managers and their practices.

Second, these experiences illuminate the range of personal, professional and contextual (organizational, health system and societal) factors that influence current managerial practice, and point to the particular influence of professional identity (see Figure 2). The current largely facility-focused management practice seems to reflect both the strong nursing identity of the managers as well as broader organizational factors. Although nurse training supports the managers in some aspects of their jobs, it appears that it does not adequately prepare them for the more strategic tasks or for their leadership of others.

Third, three of the eight managers do, nonetheless, appear to demonstrate an emerging leadership identity. Unlike their colleagues, they self-identify their roles as extending beyond clinical practice and their managerial practices are oriented more towards broader problem-solving as well as management of staff and relationships. These three managers seem, therefore, to have begun the transition from nurse to leader that theory suggests is part of, and important in, developing leadership competencies. As summarized in Figure 2, the participating FMs’ experiences also suggest that the factors influencing identity transition are both personal (such as childhood experiences and personal career aspirations) and organizational, such as previous experience of managerial roles, support for leadership and management development and broader managerial imperatives and structures. In this context, weaknesses in all these organizational factors, including managerial imperatives experienced as disempowering, seem to undermine leadership development.

Fourth, supported by wider literature (Dovey 2002; Hartley and Hinksman 2003; Dorros 2006), the participating managers offered some tentative ideas about how better to support the development of managerial and leadership competencies among PHC FMs in this context. They suggested that there is a need to:

- identify potential leaders early in their careers, those nurses with the inclination and interest to become FMs, and to provide them with exposure to, and experience of, management in advance of any formal appointment;
- provide newly appointed managers with a formal induction programme, addressing the formal components of the job description, accompanied by peer mentorship to support the sharing of tacit knowledge and support for routine problem solving;
- encourage continued informal, peer-to-peer support to share and learn from experiences, perhaps through ‘buddy systems’ or peer-led meetings;
- support personal reflective practice, to develop the self-awareness and self-confidence needed to lead, inspire and motivate their staff, perhaps through personal journals or by re-structuring existing formal meetings to provide reflection and learning spaces.
